# B‐mode ultrasound and contrast‐enhanced ultrasound for evaluation of pneumonia: A pictorial essay

**DOI:** 10.1002/ajum.12332

**Published:** 2023-02-15

**Authors:** Ehsan Safai Zadeh, Amjad Alhyari, Johannes Kroenig, Christian Görg, Corinna Trenker, Christoph F. Dietrich, Hajo Findeisen

**Affiliations:** ^1^ Interdisciplinary Centre of Ultrasound Diagnostics, Gastroenterology, Endocrinology, Metabolism and Clinical Infectiology University Hospital Giessen and Marburg, Philipps University Marburg Marburg Germany; ^2^ Department of Pulmonary and Critical Care Medicine University Hospital Giessen and Marburg, Philipps University Marburg Marburg Germany; ^3^ Haematology, Oncology and Immunology University Hospital Giessen and Marburg, Philipps University Marburg Marburg Germany; ^4^ Department Allgemeine Innere Medizin (DAIM) Kliniken Hirslanden Bern Bern Switzerland; ^5^ Interdisciplinary Centre of Ultrasound Diagnostics University Hospital Giessen and Marburg, Philipps University Marburg Marburg Germany

**Keywords:** CEUS, diagnosis, perfusion pattern, pneumonia, ultrasound

## Abstract

Due to their often peripheral pleural‐based location, pneumonias can be visualised by B‐mode ultrasound. Therefore, sonography can be used as an alternative imaging modality to chest X‐ray in suspected cases of pneumonia. Depending on the clinical background of the patient, and various underlying pathological mechanisms, a heterogeneous pattern of pneumonia is seen in both B‐mode lung ultrasound and contrast‐enhanced ultrasound. Here, we describe the spectrum of sonographic manifestations of pneumonic/inflammatory consolidation on B‐mode lung ultrasound and contrast‐enhanced ultrasound.

## Introduction

Radiological examination, including chest X‐ray and computed tomography, are the conventional imaging techniques for the evaluation of pulmonary pathology.^1^, ^2^, ^3^, ^4^ Despite known limitations, such as high interobserver variability, interdevice variability, absence of overview, and inability to visualise the central lung areas, transcutaneous lung ultrasound (LUS) can be used in clinical practice as point‐of‐care sonography for the evaluation of pleural‐based lung pathologies in the emergency department,^5^, ^6^ in intensive care units,^6^, ^7^ in palliative care units,^8^ in paediatrics,^9^ in ambulance settings^10^ and generally in outpatient care.^11^ Here, the LUS functions as the physician's new “stethoscope”.^12^, ^13^ Indeed, LUS is currently the standard procedure in the detection of pleural effusion.^14^, ^15^, ^16^, ^17^, ^18^

The diagnosis of pneumonia is made clinically, taking into account the patient's medical history and possible underlying risk factors.^19^ The imaging method is only of secondary importance for diagnostic verification. For the imaging diagnosis of community‐acquired pneumonia (CAP), LUS has a diagnostic accuracy at least equal to conventional chest X‐ray and is recommended in guidelines as an alternative procedure for the diagnosis of pneumonia.^20^, ^21^, ^22^, ^23^ Pneumonia is present in 38% of cases where the patient presents with dyspnoea and can be diagnosed by ultrasound.^24^ A distinction must be made between CAP and the heterogeneous clinical picture of hospital‐acquired pneumonia (HAP). In the clinical inpatient setting, LUS can be used at the bedside in both CAP and HAP in addition to standard imaging for short‐term follow‐up, predominantly in cases where the course of the disease is atypical, there are complications, clinically absent regression, and possibly needing diagnostic confirmation by ultrasound‐guided biopsy.^25^, ^26^

**Figure 1:**
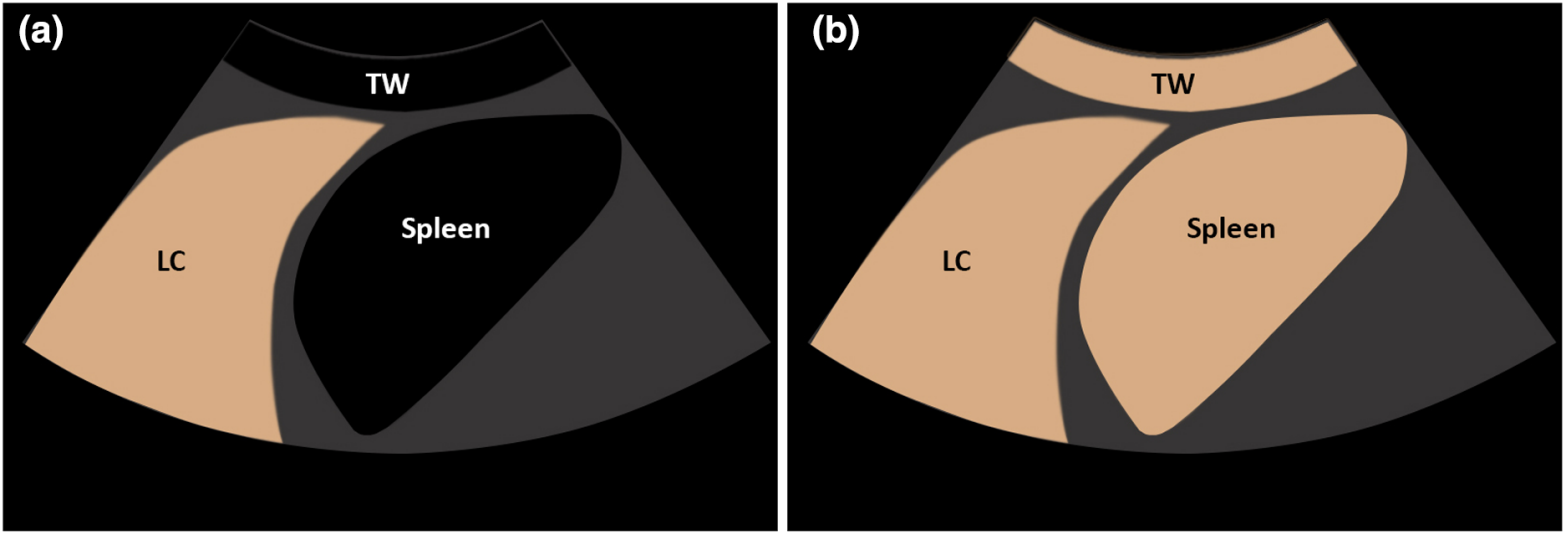
(a) Graphical illustration of early pulmonary‐arterial enhancement in a lung consolidation (LC) in comparison with the enhancement of thoracic wall (TW) and spleen. On contrast‐enhanced ultrasound, the LC reveals a complete contrast enhancement before the beginning of systemic enhancement of TW and, spleen. (b) Graphical illustration of delayed bronchial‐arterial enhancement in a LC in comparison with the enhancement of TW and spleen. On contrast‐enhanced ultrasound LC reveals a contrast enhancement simultaneous with systemic enhancement of the TW and spleen.

**Figure 2:**
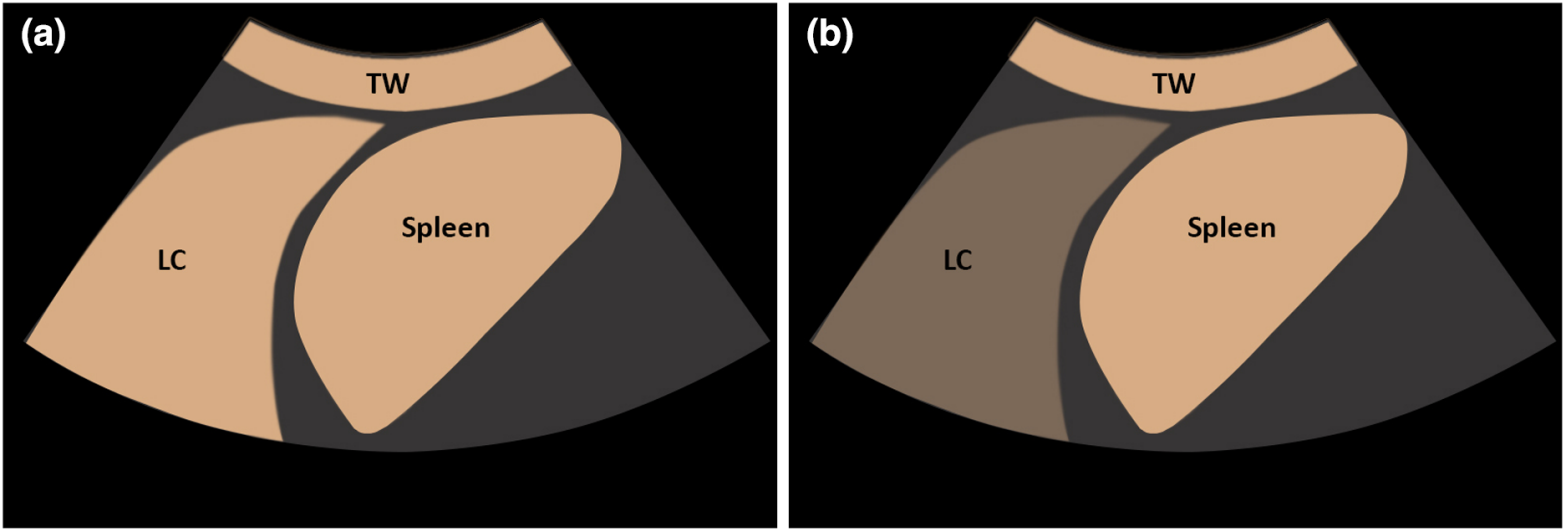
(a) Graphical illustration of marked extent of enhancement on contrast‐enhanced ultrasound in a lung consolidation (LC) in comparison with enhancement of the thoracic wall (TW) and spleen. (b) Graphical illustration of reduced extent of enhancement on contrast‐enhanced ultrasound in a LC in comparison with enhancement of the TW and spleen.

**Figure 3:**
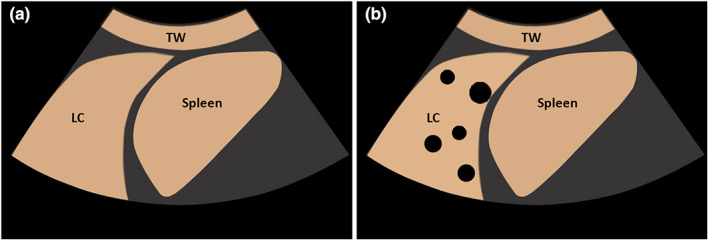
(a) Graphical illustration of homogeneous enhancement by contrast‐enhanced ultrasound in a lung consolidation (LC) in comparison with enhancement of the spleen. (b) Graphical illustration of inhomogeneous enhancement with non‐perfused areas by contrast‐enhanced ultrasound in a LC in comparison with enhancement of the spleen.

**Figure 4:**
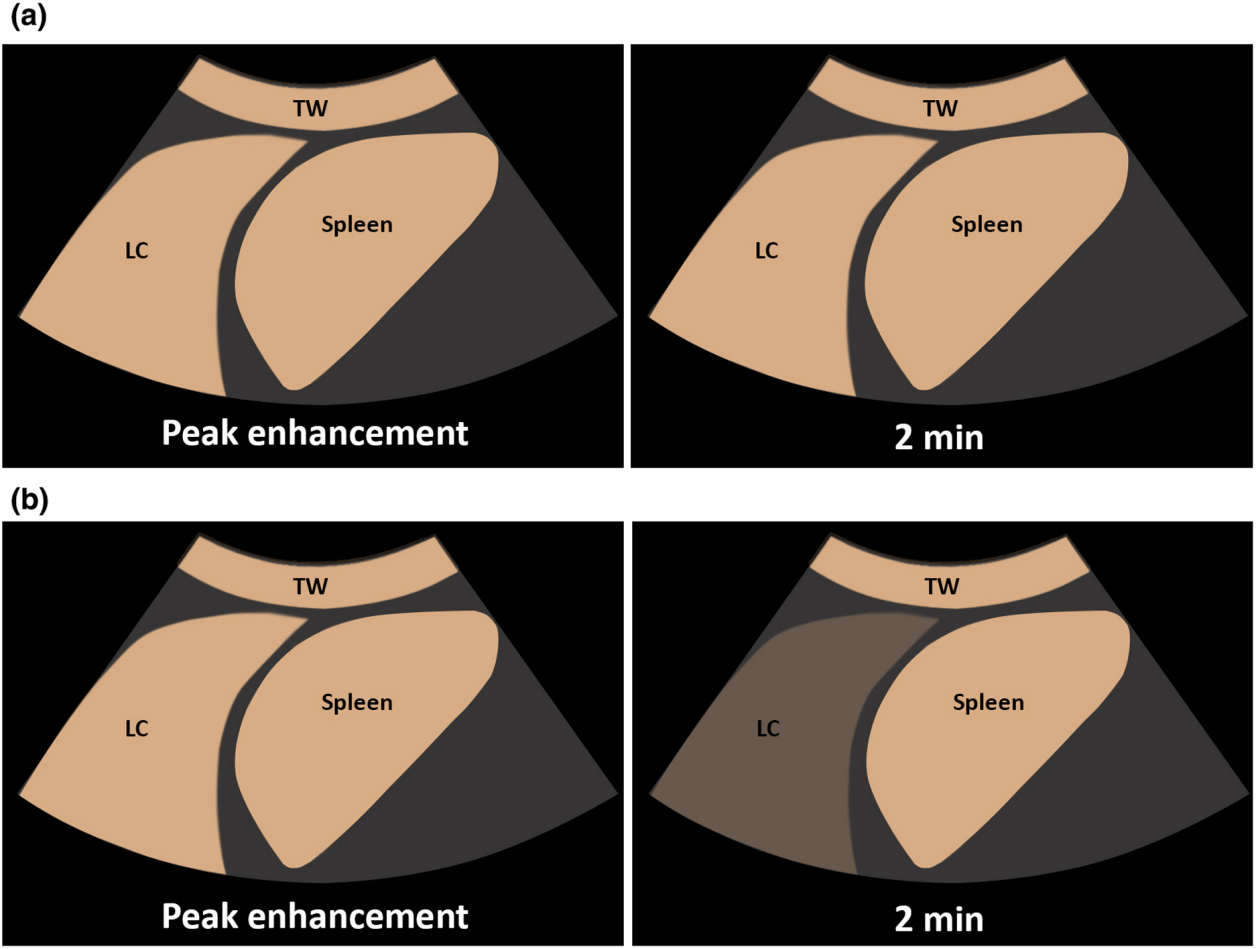
(a) Graphical illustration of late washout of enhancement on contrast‐enhanced ultrasound in a lung consolidation (LC). On contrast‐enhanced ultrasound (CEUS), the LC reveals no decrease of enhancement after 120 s in comparison with peak of enhancement of the LC (late wash‐out) (b) Graphical illustration of early washout of enhancement on contrast‐enhanced ultrasound in a LC. On CEUS, the LC reveals a marked decrease of enhancement after 120 s in comparison with peak of enhancement of the LC (early wash‐out).

B‐mode LUS patterns of pneumonia have been adequately described.^27^ CEUS enables the additional evaluation of perfusion patterns of pneumonia in terms of type of enhancement (pulmonary arterial vs. bronchial arterial), extent of enhancement (EE; isoechogenic vs. hypoechogenic vs. absent), homogeneity of enhancement (HE; homogeneous vs. inhomogeneous), and decrease of enhancement (DE; early washout [<120 s] vs. late washout [>120 s]).^28^, ^29^, ^30^ In this regard, contrast‐enhanced ultrasound (CEUS) can provide valuable information in addition to B‐mode LUS.^30^, ^32^ The aim of this Pictorial Essay is to present to the clinical practitioner the different B‐mode LUS and CEUS patterns of pneumonia according to the clinical background of the patients. The basic assessment criteria of CEUS in pulmonary pathologies in general are explained with accompanying illustrations (Figures 1, 2, 3, 4).

### Typical perfusion patterns of pneumonia on CEUS

**Figure 5:**
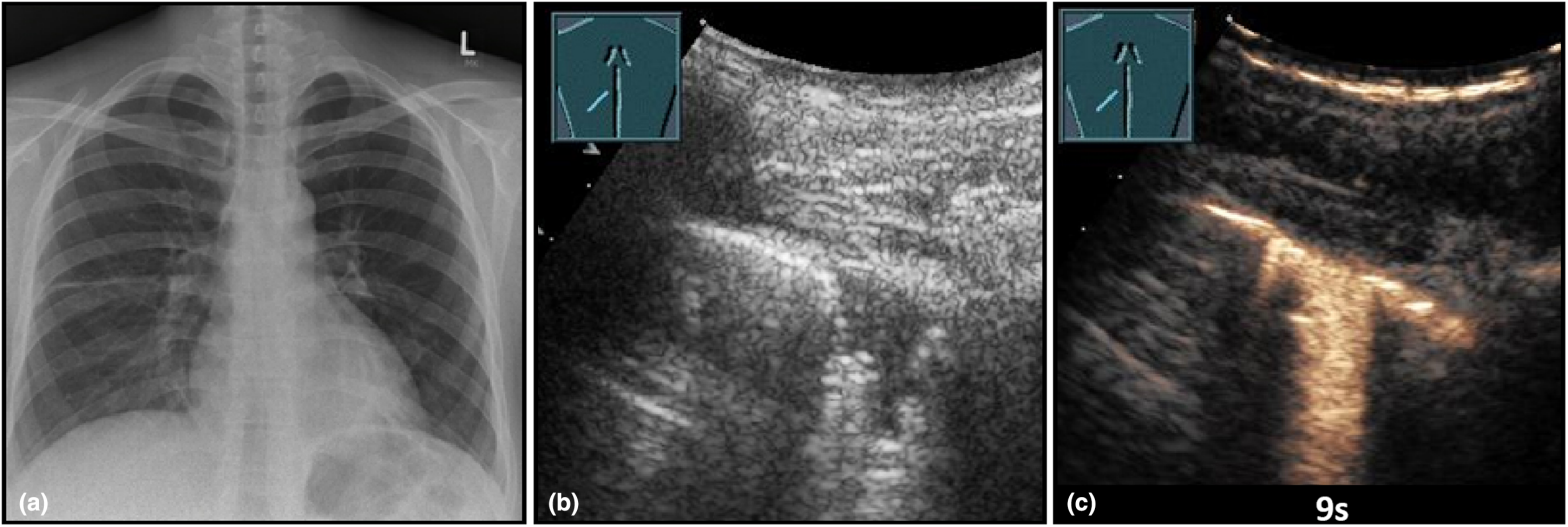
A 30‐year‐old female patient presented to the emergency department with respiration‐dependent chest pain and fever. (a) Visualisation of an unremarkable chest X‐ray (courtesy of Prof. Dr. Andreas H. Mahnken, Department of Radiology, University Hospital Marburg); (b) B‐mode ultrasound shows a hypoechoic, wedge‐shaped, pleural consolidation; (c) on contrast‐enhanced ultrasound, the lesion shows a pulmonary arterial, homogeneous perfusion pattern of enhancement, consistent with community‐acquired pleurisy.

**Figure 6:**
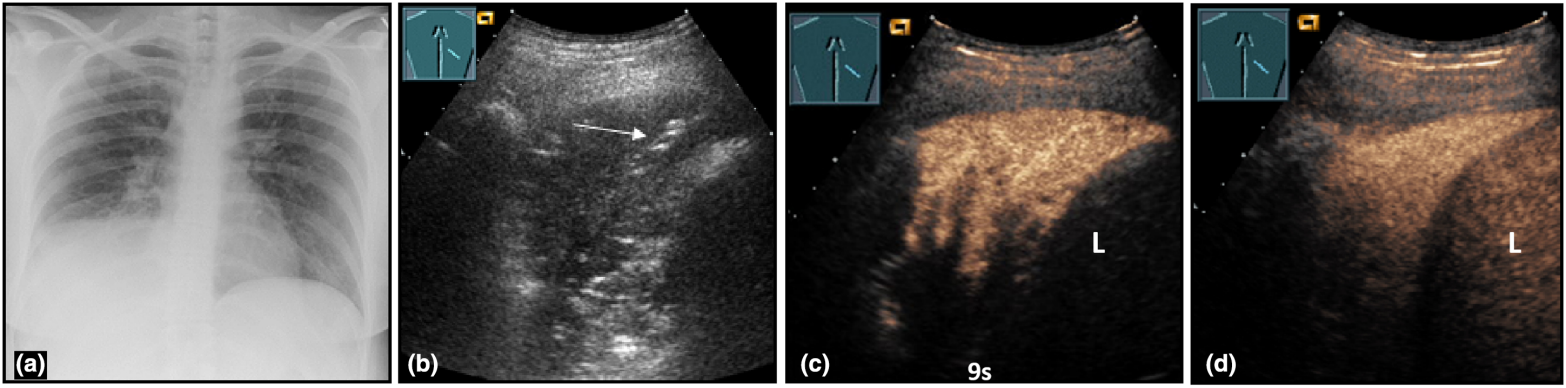
A 36‐year‐old female patient presented to the emergency department with cough, sputum, fever, and tachycardia. Visualisation of a right‐sided, low‐field consolidation on chest X‐ray (courtesy of Prof. Dr. Andreas H. Mahnken, Department of Radiology, University Hospital Marburg) (a); B‐mode ultrasound (b) shows a hypoechoic consolidation with air bronchogram (arrow); on contrast‐enhanced ultrasound, compared with the liver (L), after 9 s (c) the lesion shows a pulmonary arterial perfusion pattern, with a marked homogeneous enhancement after 2 min (d), consistent with community‐acquired pneumonia.

**Figure 7:**
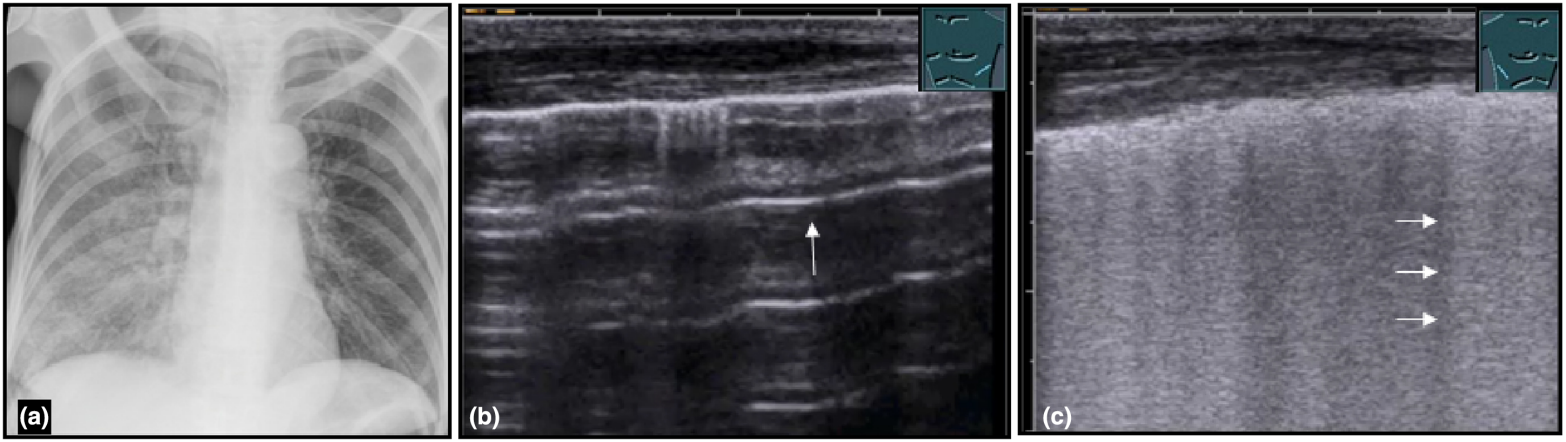
A 57‐year‐old female patient presented to the emergency department with weakness and a dry irritable cough. A chest X‐ray (courtesy of Prof. Dr. Andreas H. Mahnken, Department of Radiology, University Hospital Marburg) (a) shows a right‐sided infiltration of the lung; B‐mode ultrasound shows the healthy left lung (b), with a smooth, pleural entry echo and multiple A‐lines (arrow); and a right‐sided lung (c) with a reflexogenic lung with an irregular pleural entry echo and multiple B‐lines (arrows), consistent with community‐acquired interstitial pneumonia.

The diagnosis of pneumonia is based on the clinical symptoms of the patient (fever, cough, or dyspnea) and pathological changes in laboratory parameters (increase in inflammation parameters).^33^ Pneumonia can be differentiated into intra‐alveolar pneumonia (pleuropneumonia, lobar pneumonia)^34^, ^35^ (Figures 5 and 6) and interstitial pneumonia (Figure 7).

Patients with pleuropneumonia – focal peripheral pneumonia with simultaneous pleurisy ‐ present with respiratory localised pleural pain.^34^ Pleuropneumonia is characterised by a peripheral pleura‐based consolidation on B‐mode LUS, with homogeneous pulmonary‐arterial enhancement on CEUS (Figure 5).^34^

Patients with lobar pneumonia usually have a poor general state. Lobar pneumonia is characterised by a diffuse consolidation with or without air bronchogram on B‐mode LUS and homogeneous pulmonary‐arterial enhancement on CEUS (Figure 6).^30^, ^31^

Interstitial pneumonias are caused by viruses (*e.g*. coronavirus SARS‐CoV‐2) or by bacteria (*e.g*. mycoplasma, chlamydiae, coxiellae).^36^, ^37^ Furthermore, in patients with pneumonia symptoms, the presence of an irregular pleural line with multiple B‐lines (interstitial syndrome) on B‐mode LUS may indicate interstitial pneumonia (Figure 7).^36^ Due to the absence of consolidations, CEUS examination is not indicated.

**Figure 8:**
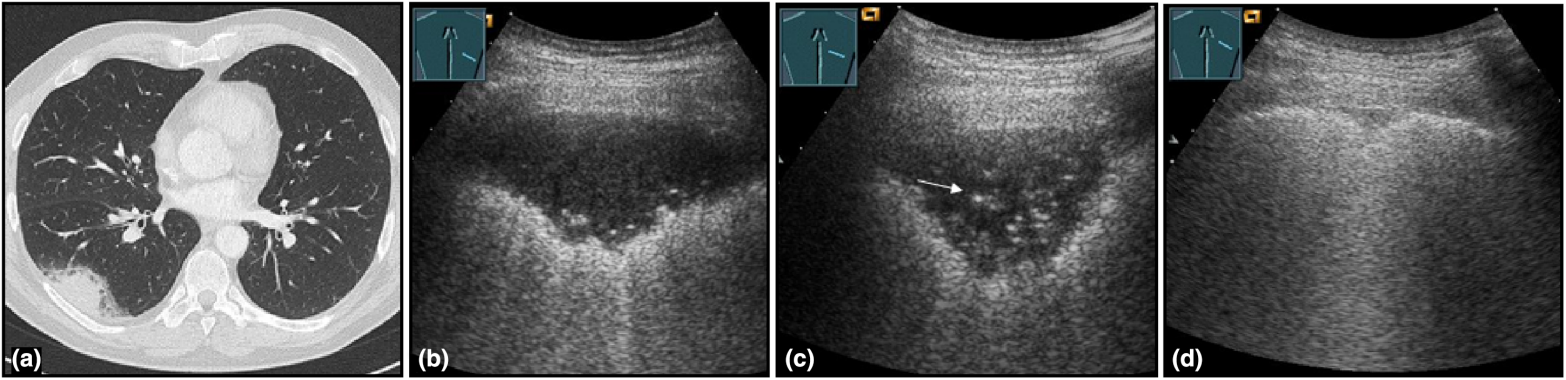
A 51‐year‐old patient presented to the emergency department with shortness of breath and respiratory pain. Computed tomography was performed to rule out pulmonary artery embolism. The computed tomography scan (courtesy of Prof. Dr. Andreas H. Mahnken, Department of Radiology, University Hospital Marburg) (a) shows a right‐sided dorsal consolidation; B‐mode ultrasound shows a homogeneous, hypoechoic consolidation (b), which regressed in size after 4 days with increasing air bronchogram (arrow) (c), and with almost complete regression after 4 weeks (d), compatible with community‐acquired pneumonia.

**Figure 9:**
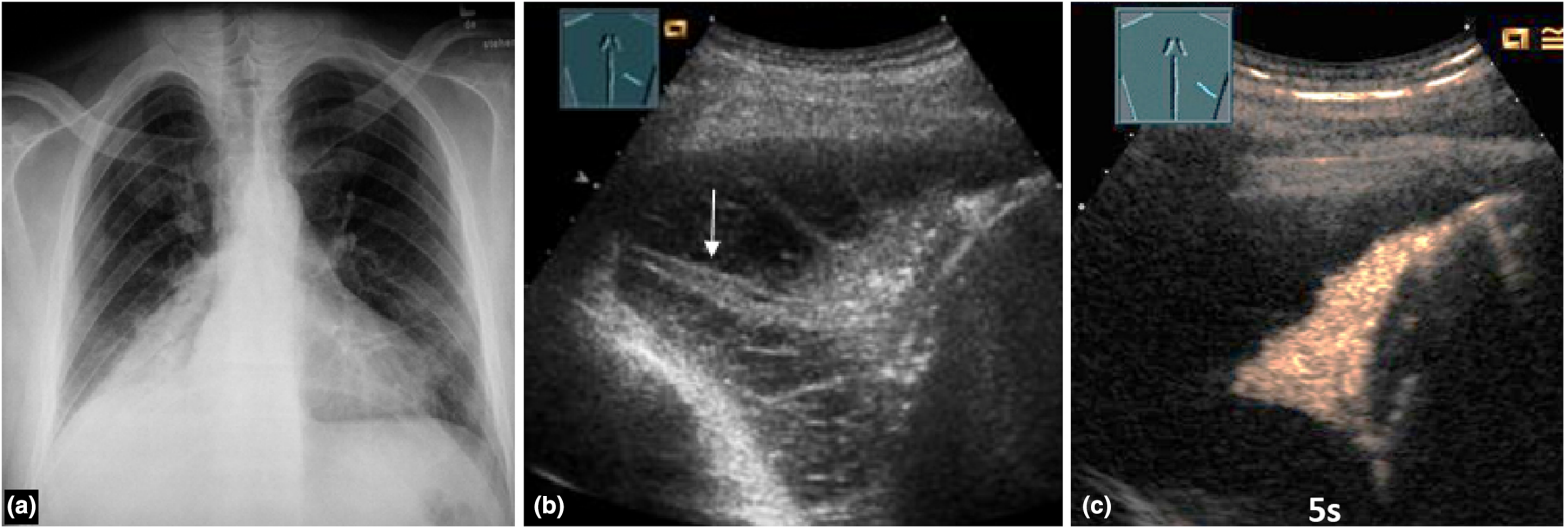
A 66‐year‐old patient presented to the emergency department with severe dyspnea, right thoracic pain, severe cough, and yellowish sputum with hemoptysis, and with subfebrile temperatures. Visualisation of a right‐sided, paracardial consolidation on chest X‐ray (courtesy of Prof. Dr. Andreas H. Mahnken, Department of Radiology, University Hospital Marburg) (a); B‐mode ultrasound (b) reveals a hypoechoic consolidation with central air bronchogram and multiple fibrin septa (arrow); on contrast‐enhanced ultrasound (c), the consolidation after 5 seconds demonstrates a homogeneous, pulmonary arterial pattern of enhancement, where the fibrin threads show an absence of enhancement consistent with pneumonia with parainfectious polyseptated effusion. Thoracoscopic pleural decortication was performed.

**Figure 10:**
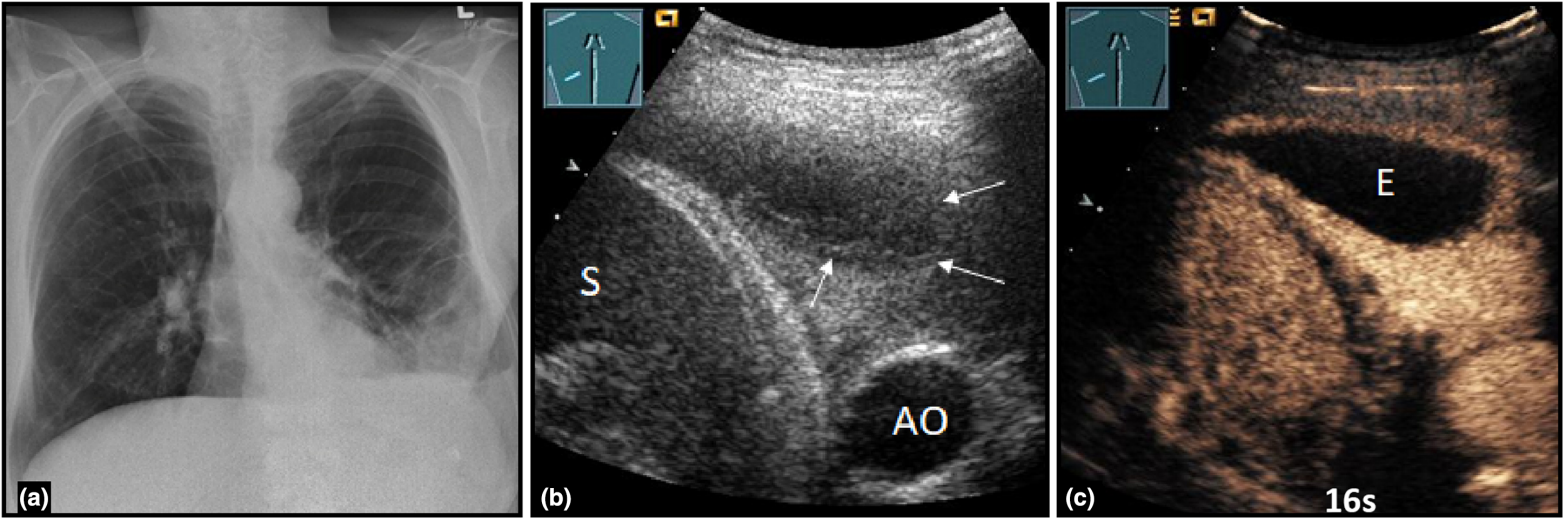
A 62‐year‐old female patient presented to the emergency department with shortness of breath and left‐sided flank pain. Presentation of a left‐sided subfield consolidation on the thoracic X‐ray (courtesy of Prof. Dr. Andreas H. Mahnken, Department of Radiology, University Hospital Marburg) (a); on B‐mode ultrasound (b), a hypoechoic consolidation with an oval, pleural, nearly anechoic area (arrows) is seen (AO = aorta, S = spleen); on contrast‐enhanced ultrasound (c), the consolidation shows a homogeneous perfusion pattern of enhancement after 16 seconds. Furthermore, a pleural‐based oval area with absent enhancement and marked wall enhancement is present, consistent with community‐acquired pneumonia with pleural empyema (E). A diagnostic/therapeutic thoracentesis of the empyema was performed.

**Figure 11:**
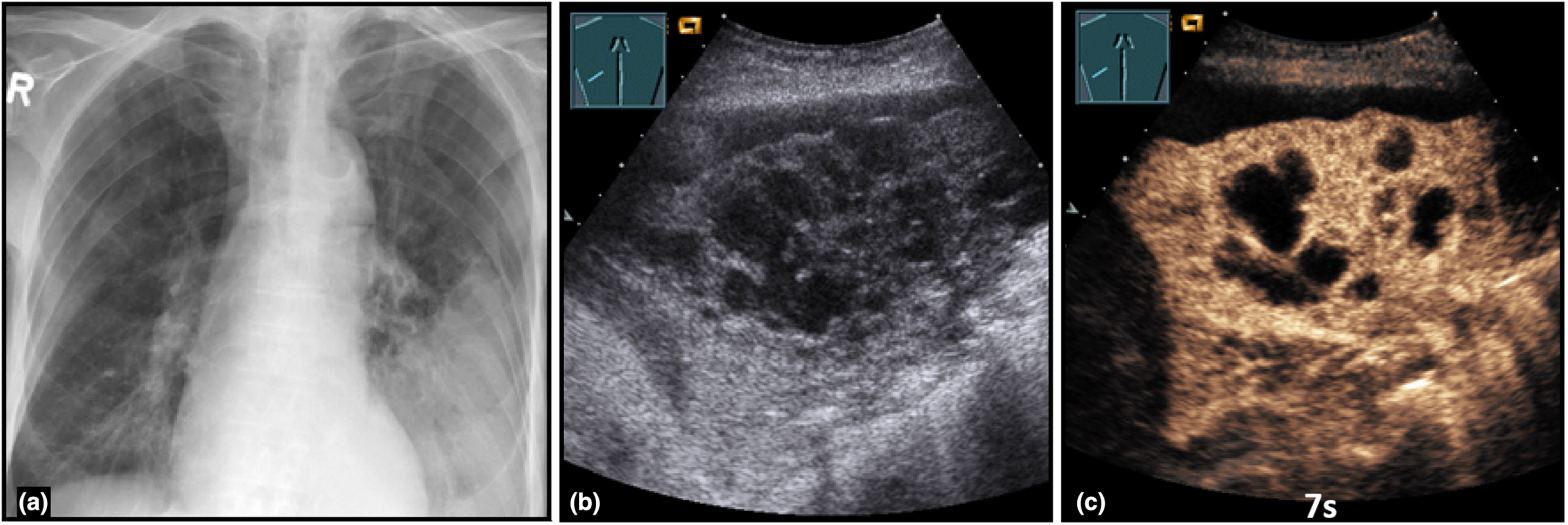
An 84‐year‐old patient with cachexia, general weakness, dry cough, and purulent sputum presented to the emergency department. Presentation of a left‐sided, low‐field consolidation on chest X‐ray (courtesy of Prof. Dr. Andreas H. Mahnken, Department of Radiology, University Hospital Marburg) (a); B‐mode ultrasound (b) shows a complex, hypoechoic consolidation with central, anechoic, confluent areas; contrast‐enhanced ultrasound (c) reveals an inhomogeneous, pulmonary arterial pattern of enhancement after 7 seconds, with multiple anechoic areas with an absence of enhancement, consistent with community‐acquired abscessed pneumonia.

Uncomplicated pneumonia shows a characteristic CEUS pattern with homogeneous pulmonary‐arterial enhancement (Figure 6) and is characterised by a corresponding rapid regression under therapy (Figure 8). A parainfectious effusion (Figure 9), pleural empyema (Figure 10), or pulmonary abscess (Figure 11) indicates a complicated course and can be detected in CEUS examinations. Pleural empyema is characterised by pleural thickening with marked enhancement on CEUS (Figure 10).^18^ In the case of lung abscess, the lung consolidation shows central areas with absent enhancement (Figure 11).^26^

**Figure 12:**
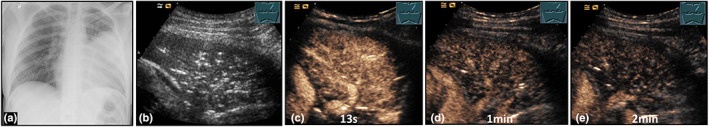
A 30‐year‐old female patient presented to the emergency department with mild dyspnea, cough, and weakness. Left‐sided consolidation was seen on chest X‐ray (courtesy of Prof. Dr. Andreas H. Mahnken, Department of Radiology, University Hospital Marburg) (a); B‐mode ultrasound (b) shows a complex, hypoechoic consolidation with a marked air bronchogram; contrast‐enhanced ultrasound shows a homogeneous, pulmonary arterial pattern of enhancement after 13 seconds (c), with early and marked washout after 1 minute (d) and 2 minutes (e), consistent with pulmonary arterial vasoconstriction. The findings are suggestive of community‐acquired lobar pneumonia.

**Figure 13:**
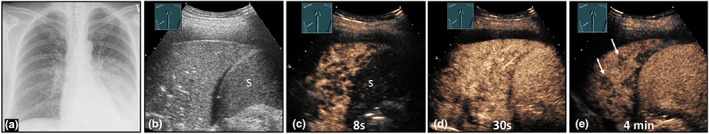
A 51‐year‐old smoker presented to the emergency department with fever, chills, dyspnea, and chest pain. Left‐sided consolidation is shown on chest X‐ray (courtesy of Prof. Dr. Andreas H. Mahnken, Department of Radiology, University Hospital Marburg) (a); B‐mode ultrasound (b) shows a homogeneous, hypoechoic, flat consolidation (S = spleen); contrast‐enhanced ultrasound reveals an inhomogeneous, pulmonary arterial pattern of enhancement after 8 seconds (c), whereas, after 30 seconds (d), the lung shows a widely homogeneous enhancement, possibly due to bronchial arterial perfusion; after 4 minutes (e), hypoechoic areas (arrows) are demarked, indicating community‐acquired pneumonia with primarily impaired, pulmonary arterial perfusion.

**Figure 14:**
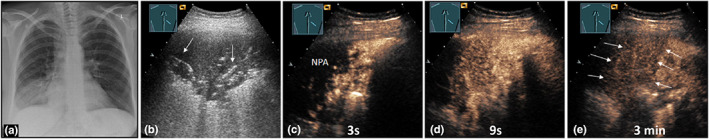
A 37‐year‐old female patient presented to the emergency department with hemoptysis and fever. Right‐sided consolidation was seen on chest X‐ray (courtesy of Prof. Dr. Andreas H. Mahnken, Department of Radiology, University Hospital Marburg) (a); B‐mode ultrasound (b) shows an inhomogeneous, hypoechoic consolidation with signs of an air bronchogram (arrows); on contrast‐enhanced ultrasound, the consolidation shows an inhomogeneous, pulmonary arterial pattern of enhancement after 3 seconds (c), with areas showing no pulmonary arterial perfusion (NPA = non‐perfused area); after 9 seconds (d), the primary NPA tissue of the lung shows a secondary bronchial arterial perfusion, which is still demarked as a hypoechoic area after 3 minutes (arrows) (e), indicating a community‐acquired pneumonia with primary partial absence of pulmonary arterial perfusion.

**Figure 15:**
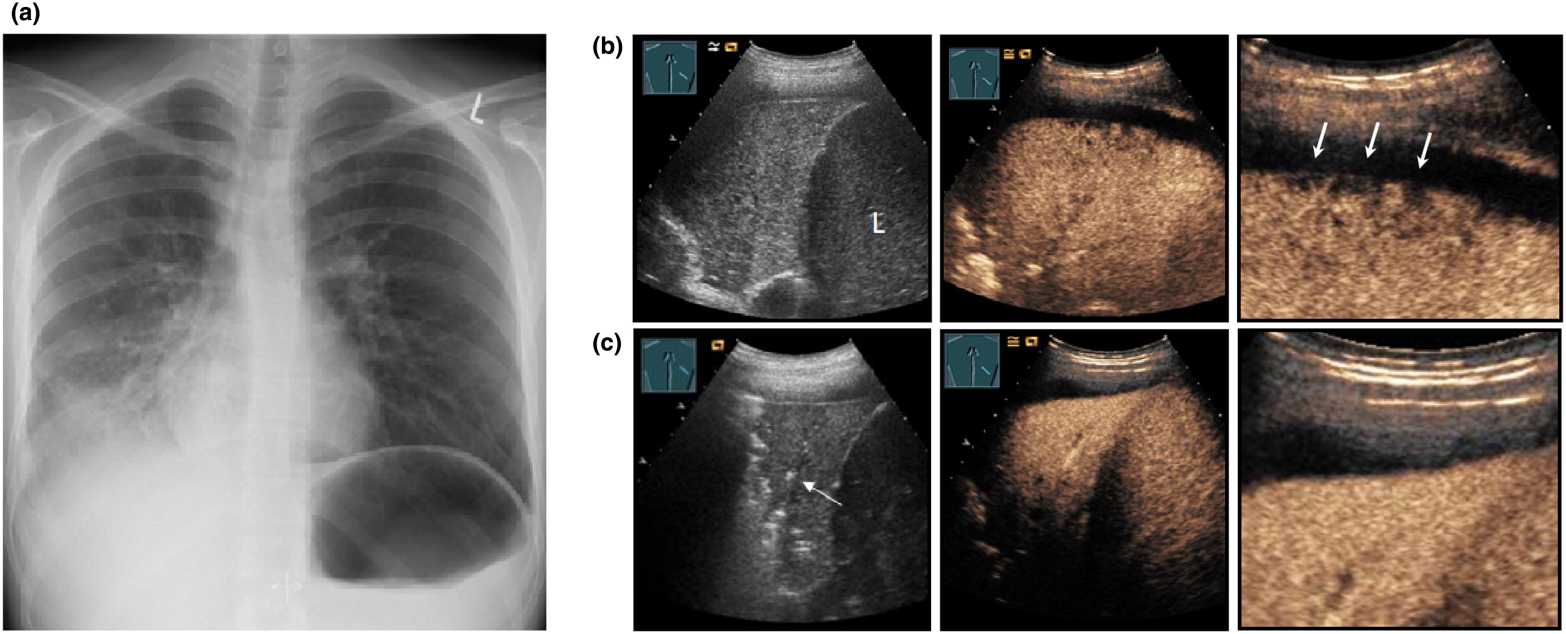
A 21‐year‐old patient with fever, loss of appetite, and weakness presented to the emergency department. (a) Presentation of a right‐sided consolidation on chest X‐ray (courtesy of Prof. Dr. Andreas H. Mahnken, Department of Radiology, University Hospital Marburg); Series (b) B‐mode ultrasound shows an inhomogeneous, hypoechoic consolidation (L = liver); on contrast‐enhanced ultrasound, after 2 minutes, the consolidation reveals a peripheral, moth‐like pleural area without contrast enhancement in overview and magnification (arrows); Series (c) after 2 days, there is regression of the consolidation with a mild air bronchogram (arrow); furthermore, a complete reperfusion of the peripheral non‐perfused areas can be observed, indicating a community‐acquired pneumonia with primarily disturbed, peripheral, pulmonary arterial and bronchial arterial perfusion. Final diagnosis was pneumonia due to *Mycoplasma pneumoniae*.

In CEUS, further various pulmonary‐arterial perfusion disturbances associated with pneumonia can be observed (Figures 12, 13, 14, 15). The lesions may show an early and marked washout (Figure 12). Furthermore, the lesions may reveal areas of absent perfusion during the pulmonary‐arterial phase with present (Figures 13 and 14) or absent perfusion (Figure 15) during the bronchial‐arterial phase. These patterns may be hypoxemia‐related due to the Euler–Liljestand mechanism.^38^ In patients with pneumonia, local hypoxia may cause reflex vasoconstriction of pulmonary‐arterial vessels, with an increase of local flow resistance.^39^ In the event of adequate systemic bronchial‐arterial supply (in patients with sufficient cardiac function), pneumonic consolidation can be perfused by the bronchial artery (Figures 13 and 14). In the case of inadequate systemic bronchial‐arterial supply (in patients with inadequate cardiac function), the absence of pulmonary‐arterial supply cannot be compensated by the bronchial artery (Figure 15).^38^ However, the definite underlying pathogenesis and clinical relevance of these perfusion disturbances is still unclear.

### Contrast‐enhanced ultrasound pattern of pneumonia with specific pathogenesis

**Figure 16:**
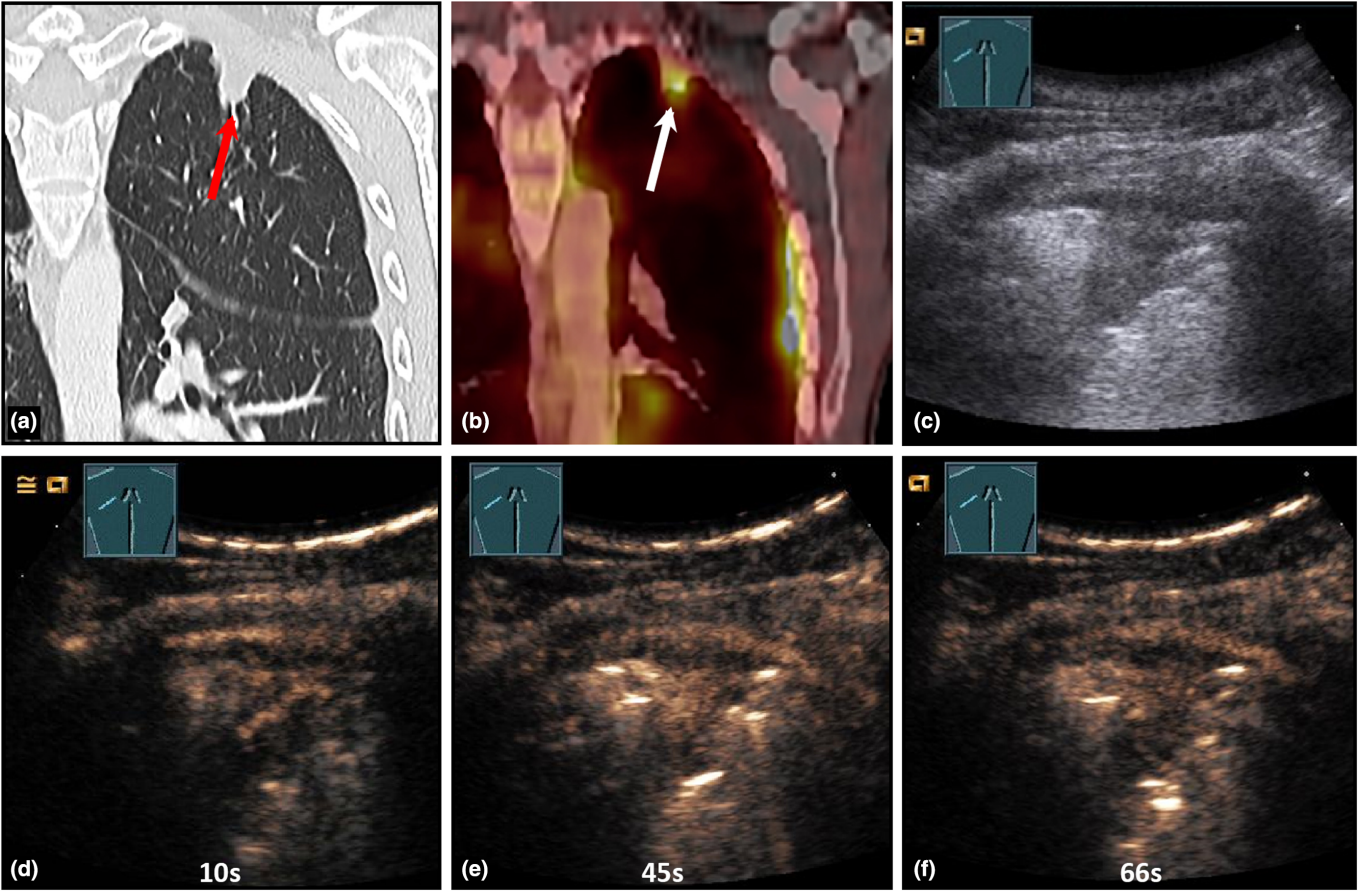
A 39‐year‐old female patient was admitted with unexplained left‐sided pleural effusions and respiratory pain. The computed tomography (courtesy of Prof. Dr. Andreas H. Mahnken, Department of Radiology, University Hospital Marburg) shows a lung consolidation in the left upper lobe (a). The lesions reveal an increased Fluorodeoxyglucose uptake on positron emission tomography computed tomography (courtesy of Prof. Dr. Markus Luster, Department of Nuclear Medicine, University Hospital Marburg) (b). B‐mode ultrasound shows a hypoechoic lung consolidation in the left upper lobe (c). On contrast‐enhanced ultrasound, after 10 s, the lesion shows an inhomogeneous, bronchial‐arterial pattern of enhancement with small non‐perfused areas (d); after 45 s, the lesion still shows an inhomogeneous enhancement (e); after 66 s, the lesion shows a decrease of enhancement (rapid washout) (f). Ultrasound‐guided biopsy showed a granulomatous inflammation. With a positive QuantiFERON‐TB Gold test, a diagnosis of tuberculous pleuropneumonia was made.

**Figure 17:**
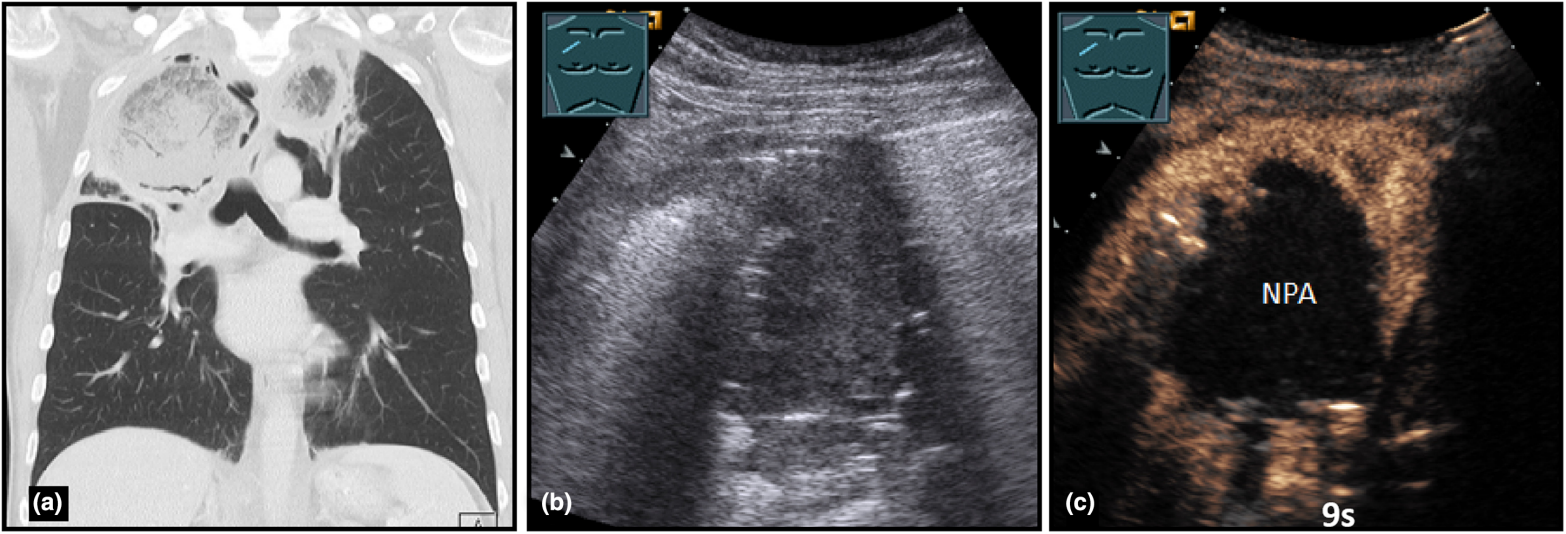
A 58‐year‐old patient with highly malignant lymphoma under aggressive polychemotherapy presented with neutropenia, dyspnea, and fever. Visualisation of a right‐sided, apical lung consolidation on computed tomography (courtesy of Prof. Dr. Andreas H. Mahnken, Department of Radiology, University Hospital Marburg) (a); B‐mode ultrasound (b) shows a hypoechoic consolidation; on contrast‐enhanced ultrasound (c), a central non‐perfused area was seen after 2 minutes, with detection of fungal hyphae in the Grocott staining. The diagnosis of fungal pneumonia in terms of invasive pulmonary mycosis was made. Surgical treatment was rejected by the patient.

**Figure 18:**
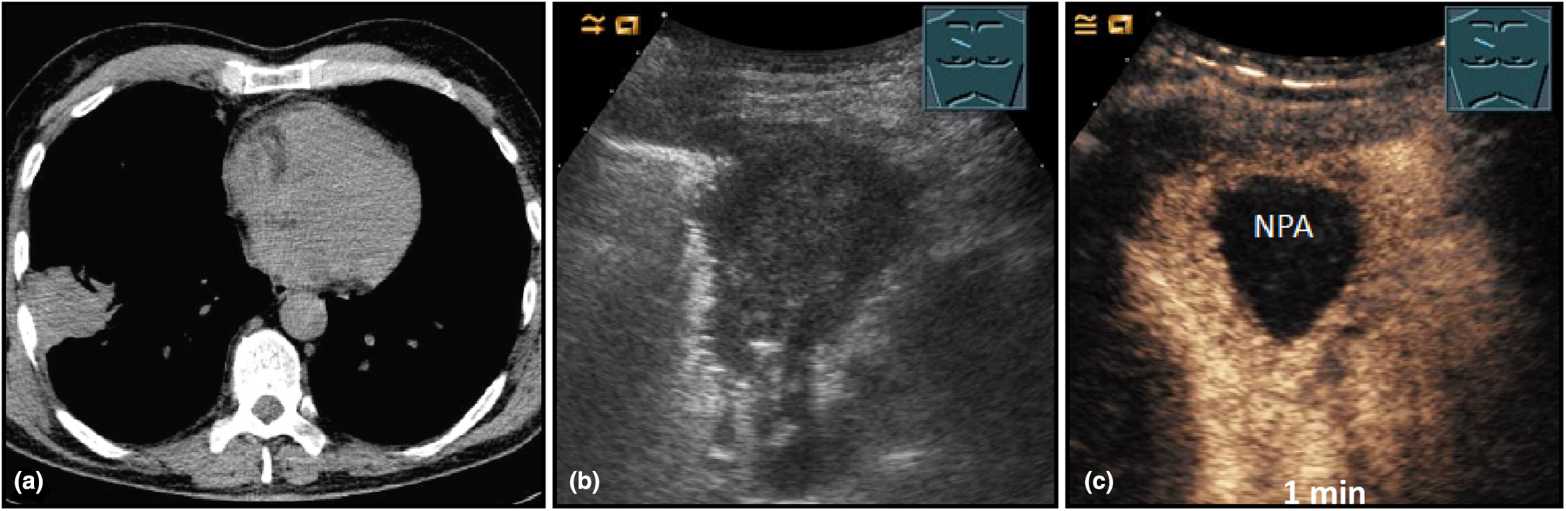
A 52‐year‐old patient with acute myeloid leukaemia under aggressive polychemotherapy, neutropenia, fever, and suspected *Aspergillus* pneumonia was treated with antifungal therapy for several weeks. An allogeneic bone marrow transplantation was planned. Visualisation of a right‐sided residual peripheral lung consolidation on computed tomography (courtesy of Prof. Dr. Andreas H. Mahnken, Department of Radiology, University Hospital Marburg) (a) after therapy; B‐mode ultrasound (b) shows a hypoechoic, homogeneous consolidation; contrast‐enhanced ultrasound (c) showed a central smooth‐bordered focal lesion with absence of enhancement (NPA = non‐perfused area) after 1 minute. A partial lung resection was performed, and histologically no more fungi could be detected in the resected tissue.

**Figure 19:**
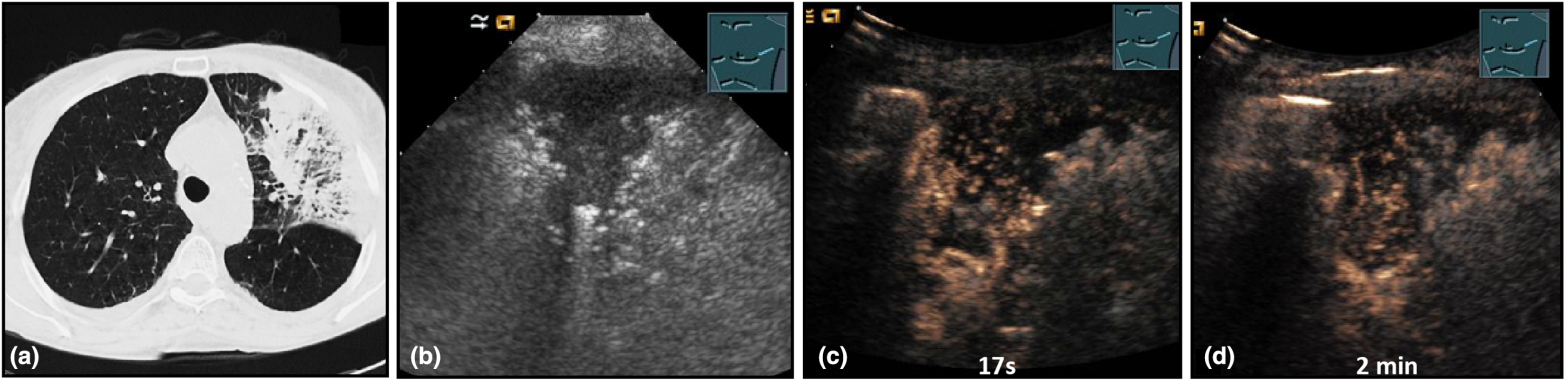
A 66‐year‐old patient presented with left‐sided bronchial carcinoma and shortness of breath and cough. The patient underwent percutaneous irradiation of the tumour region up to 50.4 Gy, with subsequent dose increase in the primary tumour area to 59.4 Gy. Computed tomography (courtesy of Prof. Dr. Andreas H. Mahnken, Department of Radiology, University Hospital Marburg) (a) shows a left‐sided peripheral lung infiltration; B‐mode ultrasound (b) shows a hypoechoic, irregularly bordered consolidation; contrast‐enhanced ultrasound reveals no pulmonary arterial perfusion. After 17 seconds (c) and after 2 minutes (d), a tender bronchial arterial perfusion can be detected, consistent with radiation pneumonitis.

**Figure 20:**
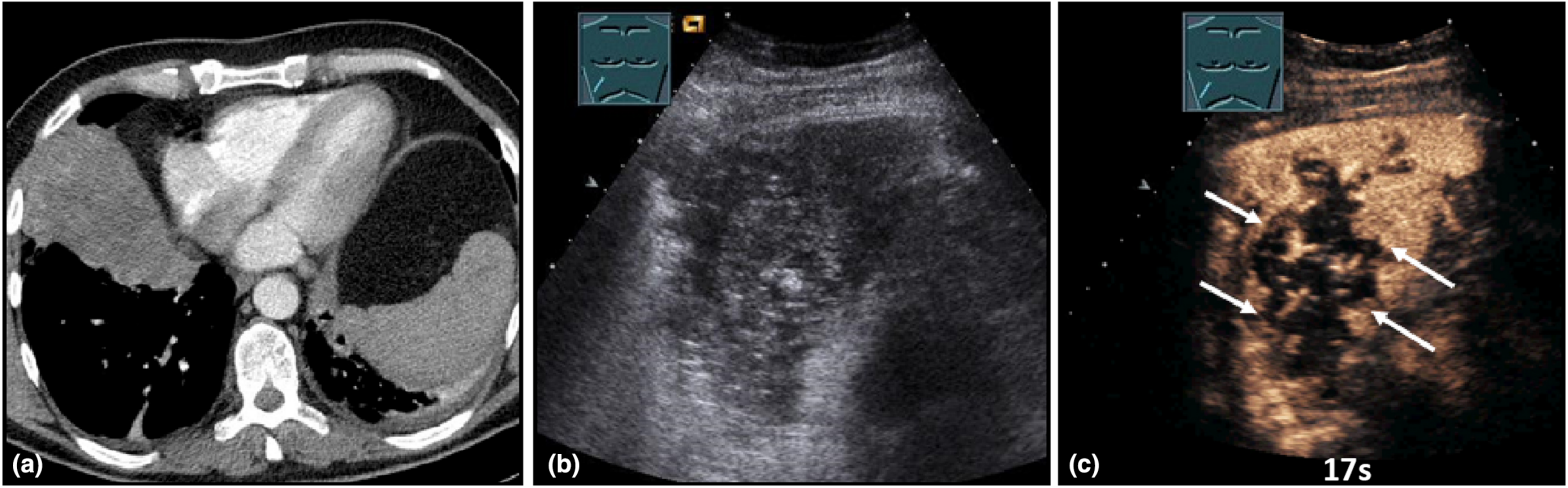
A 55‐year‐old male patient presented to the emergency department with respiratory distress due to aspiration of fire‐eater oil. Visualisation of a right‐sided, homogeneous, middle‐lobe consolidation on computed tomography (courtesy of Prof. Dr. Andreas H. Mahnken, Department of Radiology, University Hospital Marburg) (a); B‐mode ultrasound (b) shows a hypoechoic, irregularly bordered, inhomogeneous consolidation; contrast‐enhanced ultrasound (c) shows a pulmonary arterial perfusion after 17 seconds with a central non‐enhanced area (arrows), suggesting the presence of aspiration pneumonia with a complete regression after antibiotic therapy.

**Figure 21:**
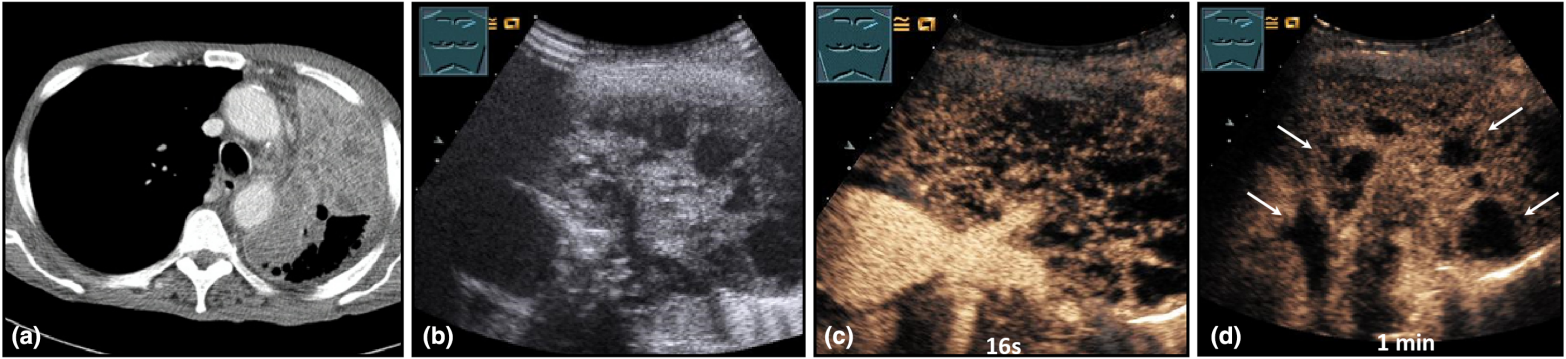
A 70‐year‐old patient presented with fever, weight loss, shortness of breath, and cough with purulent sputum due to left‐sided bronchial carcinoma. Visualisation of a left‐sided, central tumour formation with subsequent inhomogeneous consolidation on computed tomography (courtesy of Prof. Dr. Andreas H. Mahnken, Department of Radiology, University Hospital Marburg) (a); B‐mode ultrasound shows a hypoechoic, inhomogeneous consolidation (b); contrast‐enhanced ultrasound reveals pure bronchial arterial perfusion after 16 seconds (c). After 1 minute (d), anechoic, round lesions (arrows) demark, as in obstructive purulent pneumonitis. Bronchoscopic transtumoral recanalization was not successful.

The sonographic appearance of pneumonia depends on the underlying aetiology. Therefore, tuberculous pleuropneumonia shows a bronchial‐arterial hypoenhancement with areas of absent enhancement due to avascular areas (granulomas) and a rapid washout (Figure 16).^40^ In patients with a clinical or radiological suspicion of tuberculosis, this pattern may be suggestive of tuberculous pneumonia.^40^ Pulmonary candidiasis (Figure 17) and pulmonary aspergilloma (Figure 18) can also demonstrate areas of absent perfusion due to avascular areas (abscess, necrosis, haemorrhage). The patients frequently have immune suppression due to an underlying malignant disease or systemic inflammatory disease. However, the definitive diagnosis can be made only by histological confirmation.^41^ Various underlying pathomechanisms may lead to radiation pneumonitis (Figure 19), aspiration pneumonitis (Figure 20), or obstructive pneumonitis (Figure 21). The diagnosis can be made only based on the clinical background of the patient.

**Figure 22:**
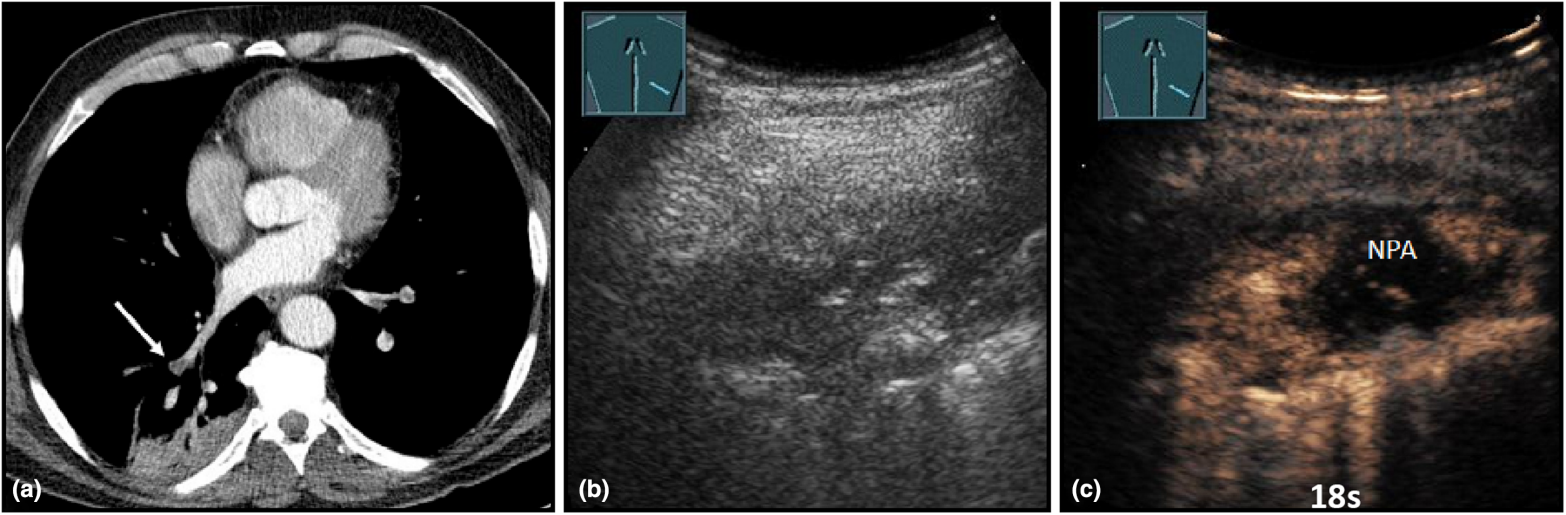
A 56‐year‐old patient was in intensive care with fever, shortness of breath, evidence of deep vein thrombosis and pulmonary artery embolism following non‐invasive ventilation, and known cardiomyopathy. Visualisation of a right‐sided, dorsal, peripheral consolidation on computed tomography (courtesy of Prof. Dr. Andreas H. Mahnken, Department of Radiology, University Hospital Marburg) (a), with central, pulmonary artery embolism (arrow); B‐mode ultrasound (b) shows a hypoechoic, inhomogeneous consolidation with air reflexes; contrast‐enhanced ultrasound (c) shows inhomogeneous enhancement with non‐perfused peripheral areas (NPA) after 18 s, indicating the presence of infarct pneumonia.

**Figure 23:**
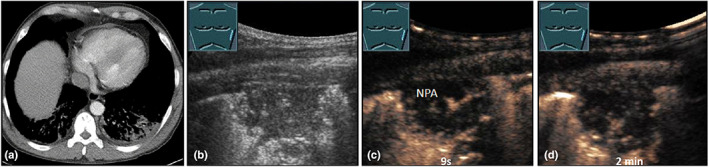
A 29‐year‐old male patient presented to the emergency department with respiratory‐dependent severe thoracic pain with fever, in the setting of known homozygous sickle cell disease. Left‐sided, dorsal, peripheral lung consolidation was seen on computed tomography (courtesy of Prof. Dr. Andreas H. Mahnken, Department of Radiology, University Hospital Marburg) (a); B‐mode ultrasound (b) shows a hypoechoic, irregularly bordered, inhomogeneous consolidation; contrast‐enhanced ultrasound shows only marginal pulmonary arterial perfusion after 9 seconds (c), with a large, central non‐perfused area also visible after 2 minutes (d), consistent with infarct pneumonia caused by sickle cell crisis.

**Figure 24:**
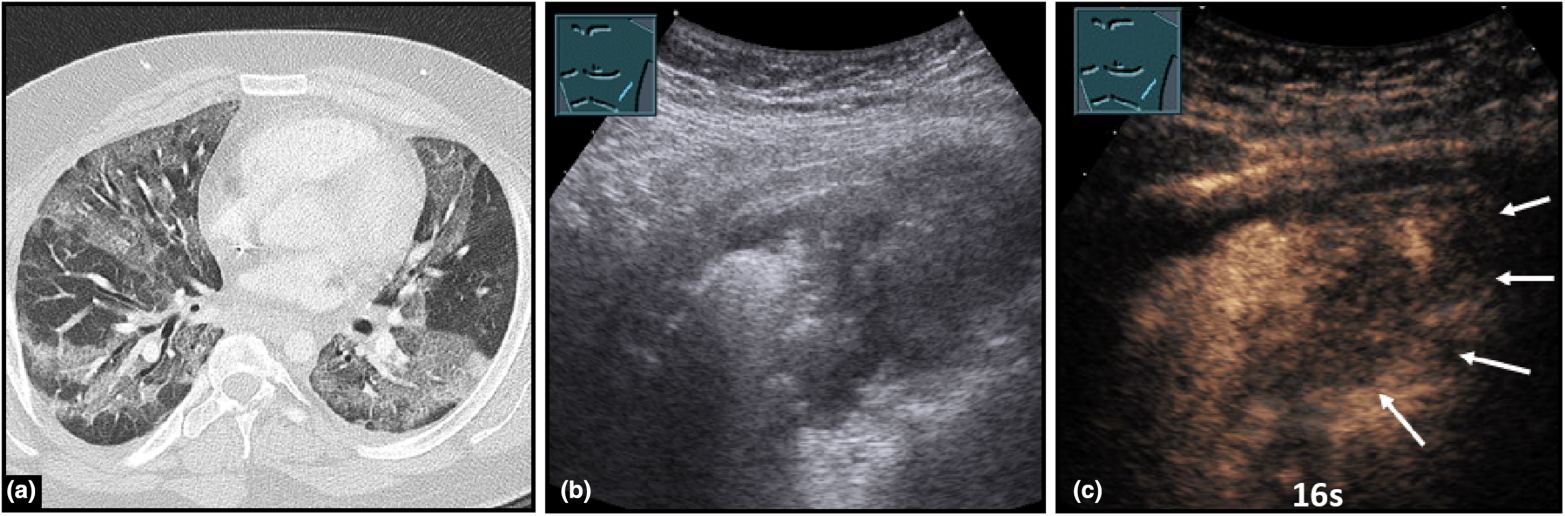
A 23‐year‐old patient presented with COVID‐19 pneumonia. Visualisation of bilateral, milky‐glass‐like pulmonary infiltrates on computed tomography (courtesy of Prof. Dr. Andreas H. Mahnken, Department of Radiology, University Hospital Marburg) (a); B‐mode ultrasound (b) shows a hypoechoic, irregularly bordered consolidation; contrast‐enhanced ultrasound (c) reveals an inhomogeneous perfusion with areas of reduced/absent perfusion (arrows) after 16 seconds, indicating the presence of COVID‐19 pneumonia with impaired perfusion.

Disturbance of the pulmonary‐arterial supply can result in pneumonic infarct (Figure 22) and is not unusual in pneumonia caused by sickle cell crisis (Figure 23).^42^ However, perfusion disturbances can also be seen in COVID‐19 pneumonia (Figure 24).^37^ The variable perfusion patterns of these specific pneumonias can be explained by the underlying pathomechanism.

### Pitfalls in the use of perfusion patterns on CEUS in the diagnosis of pneumonia

**Figure 25:**
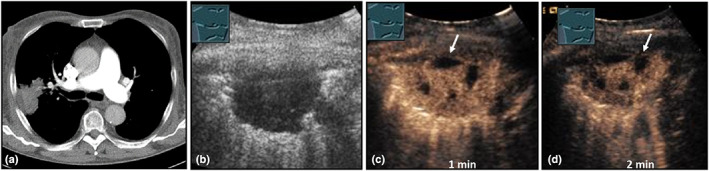
A 77‐year‐old patient presented with gastrointestinal stromal tumour under imatinib therapy, with refractory cough, bloody sputum, and known heart failure. Visualisation of a right‐sided, peripheral consolidation on computed tomography (courtesy of Prof. Dr. Andreas H. Mahnken, Department of Radiology, University Hospital Marburg) (a); B‐mode ultrasound shows a homogeneous, hypoechoic, smooth‐bordered consolidation (b); contrast‐enhanced ultrasound reveals an inhomogeneous perfusion with areas of absent perfusion (arrows) and mild washout of perfused areas after 1 min (c) and after 2 min (d). Ultrasound‐guided biopsy for suspected carcinoma revealed a diagnosis of organising pneumonia.

**Figure 26:**
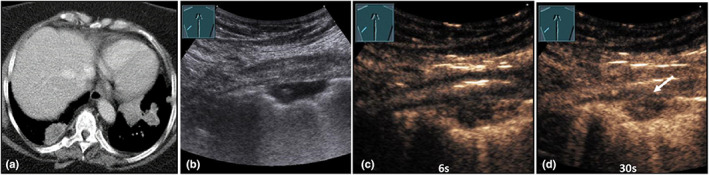
A 56‐year‐old female patient presented with fever, dyspnea, ear pain, weight loss, and shortness of breath without improvement after antibiotic therapy; there was laboratory evidence of positive perinuclear anti‐neutrophil cytoplasmic antibody levels on serologic examination. Computed tomography (courtesy of Prof. Dr. Andreas H. Mahnken, Department of Radiology, University Hospital Marburg) (a) shows multiple predominantly right‐sided consolidations; B‐mode ultrasound (b) shows a left‐sided, hypoechoic, pulmonary limited consolidation; contrast‐enhanced ultrasound shows reduced bronchial arterial perfusion after 6 s (c) and after 30 s (d) with areas of absent perfusion (arrow). Ultrasound‐guided biopsy revealed the diagnosis of granulomatosis with polyangiitis.

**Figure 27:**
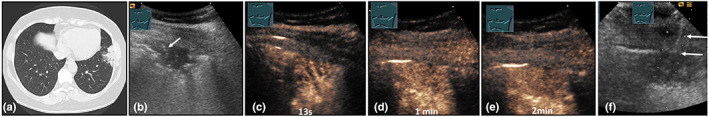
A 62‐year‐old female patient presented to the emergency department *via* a general physician with “refractory infiltrate of the lung”, with fever and cough. Visualisation of a left‐sided, pulmonary infiltrate on computed tomography (courtesy of Prof. Dr. Andreas H. Mahnken, Department of Radiology, University Hospital Marburg) (a); B‐mode ultrasound (b) shows a hypoechoic, irregularly bordered consolidation with suggested air bronchogram (arrow); contrast‐enhanced ultrasound shows homogeneous, branch‐like, pulmonary arterial perfusion after 13 s (c), with marked enhancement after 1 min (d) and after 3 min (e); in the absence of regression, ultrasound‐guided biopsy (f) (arrows) was performed, with a diagnosis of lepidic growth mucinous adenocarcinoma.

On CEUS, chronic organising pneumonia^41^ (Figure 25) and granulomatous inflammation (Figure 26)^40^ may show a bronchial‐arterial pattern of enhancement with rapid washout similar to the pattern of malignancy and are considered to be pitfalls in the diagnosis of pneumonia. The definitive diagnosis is made histologically.^40^, ^41^ Another important obstacle to diagnosis is lepidic growth adenocarcinoma, which can mimic pneumonia clinically, on B‐mode LUS and on CEUS (Figure 27).^43^

## Conclusion

**Figure 28:**
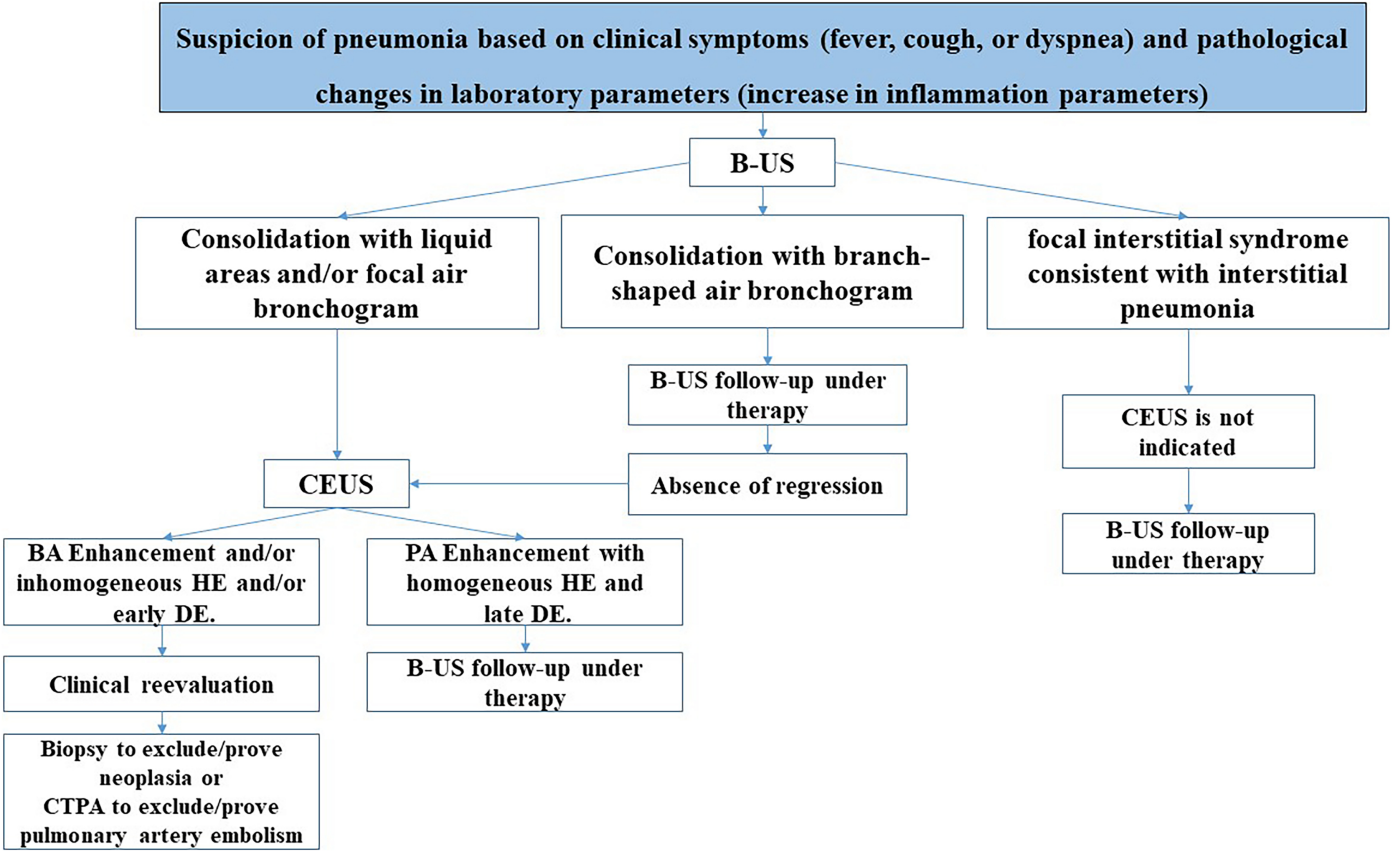
Work up strategy for evaluation of pneumonia. B‐US, B‐mode ultrasound; BA enhancement, bronchial‐arterial enhancement; CEUS, contrast‐enhanced ultrasound; CTPA, computed tomography pulmonary angiogram; DE, decrease of enhancement; HE, homogeneity of enhancement; PA enhancement, pulmonary‐arterial enhancement.

In summary, pneumonias show a heterogeneous pattern on B‐mode LUS and CEUS. Type of enhancement, extent of enhancement, homogeneity of enhancement, and decrease of enhancement are used to evaluate CEUS according to the guidelines.^28^, ^30^ Sonographic evaluation of findings is based on clinical background of patients, sonographic follow‐up, and in some cases on ultrasound‐guided histological confirmation (Figure 28). In patients with suspected pneumonia, sonography provides indicative findings for the final diagnosis.

## Funding

No funding information is provided.

## Conflict of interest

C. Görg received funding from Bracco Imaging. Bracco Imaging supported CEUS workshops at the University Hospital Marburg.

## Author contributions

**Ehsan Safai Zadeh:** Conceptualization (lead); methodology (lead); writing – original draft (lead); writing – review and editing (lead). **Amjad Alhyari:** Conceptualization (lead); methodology (lead); writing – original draft (lead); writing – review and editing (lead). **Johannes Kroenig:** Writing – review and editing (equal). **Christian Görg:** Data curation (lead); investigation (lead); methodology (lead); writing – review and editing (equal). **Corinna Trenker:** Writing – review and editing (equal). **Christoph F Dietrich:** Writing – review and editing (equal). **Hajo Findeisen:** Conceptualization (equal); methodology (equal); writing – review and editing (lead).

## References

[1] Frauenfelder T , Landsmann A . Pulmonary nodules and pneumonia: a diagnostic guideline. Radiologe 2022; 62(2): 109–19. 35020003 10.1007/s00117-021-00953-w PMC8753325

[2] Callister MEJ , Baldwin DR , Akram AR , Barnard S , Cane P , Draffan J , *et al*. British Thoracic Society guidelines for the investigation and management of pulmonary nodules: accredited by NICE. Thorax 2015; 70(Suppl 2): ii1–ii54. 26082159 10.1136/thoraxjnl-2015-207168

[3] Grief SN , Loza JK . Guidelines for the evaluation and treatment of pneumonia. Prim Care 2018; 45(3): 485–503. 30115336 10.1016/j.pop.2018.04.001 PMC7112285

[4] Detterbeck FC , Mazzone PJ , Naidich DP , Bach PB . Screening for lung cancer: diagnosis and management of lung cancer, 3rd ed: American College of Chest Physicians evidence‐based clinical practice guidelines. Chest 2013; 143(5 Suppl): e78S–92S. 23649455 10.1378/chest.12-2350 PMC3749713

[5] Volpicelli G . Point‐of‐care lung ultrasound. Praxis (Bern 1994) 2014; 103(12): 711–6. 24894615 10.1024/1661-8157/a001690

[6] Heldeweg MLA , Vermue L , Kant M , Brouwer M , Girbes ARJ , Haaksma ME , *et al*. The impact of lung ultrasound on clinical‐decision making across departments: a systematic review. Ultrasound J. 2022; 14(1): 5. 35006383 10.1186/s13089-021-00253-3 PMC8748548

[7] Kulkarni S , Down B , Jha S . Point‐of‐care lung ultrasound in intensive care during the COVID‐19 pandemic. Clin Radiol 2020; 75(9): 710.e1–4. 10.1016/j.crad.2020.05.001 PMC7218373 32405081

[8] Dhamija E , Thulkar S , Bhatnagar S . Utility and potential of bedside ultrasound in palliative care. Indian J Palliat Care 2015; 21(2): 132–6. 26009664 10.4103/0973-1075.156465 PMC4441172

[9] Yilmaz HL , Özkaya AK , Sarı Gökay S , Tolu Kendir Ö , Şenol H . Point‐of‐care lung ultrasound in children with community acquired pneumonia. Am J Emerg Med 2017; 35(7): 964–9. 28202294 10.1016/j.ajem.2017.01.065

[10] Moore CL , Copel JA . Point‐of‐care ultrasonography. N Engl J Med 2011; 364(8): 749–57. 21345104 10.1056/NEJMra0909487

[11] Sorensen B , Hunskaar S . Point‐of‐care ultrasound in primary care: a systematic review of generalist performed point‐of‐care ultrasound in unselected populations. Ultrasound J. 2019; 11(1): 31. 31749019 10.1186/s13089-019-0145-4 PMC6868077

[12] Copetti R . Is lung ultrasound the stethoscope of the new millennium? Definitely yes! Acta Med Acad 2016; 45(1): 80–1. 27284804 10.5644/ama2006-124.162

[13] Filly RA . Ultrasound: the stethoscope of the future, alas. Radiology 1988; 167(2): 400. 3282260 10.1148/radiology.167.2.3282260

[14] Jany B , Welte T . Pleural effusion in adults‐etiology, diagnosis, and treatment. Dtsch Arztebl Int 2019; 116(21): 377–86. 31315808 10.3238/arztebl.2019.0377 PMC6647819

[15] Havelock T , Teoh R , Laws D , Gleeson F . Pleural procedures and thoracic ultrasound: British Thoracic Society pleural disease guideline 2010. Thorax 2010; 65(Suppl 2): i61–76. 10.1136/thx.2010.137026 20696688

[16] Findeisen H , Trenker C , Zadeh ES , Görg C . Further aspects concering peripheral lung carcinoma in CEUS. Eur J Ultrasound 2020; 42: 323. 10.1055/a-1090-4327 32040969

[17] Safai Zadeh E , Görg C , Dietrich CF , Görlach J , Alhyari A , Trenker C . Contrast‐enhanced ultrasound for evaluation of pleural effusion: a pictorial essay. J Ultrasound Med 2022; 41(2). 10.1002/jum.15705. 33782994

[18] Safai Zadeh E , Weide J , Dietrich CF , Trenker C , Koczulla AR , Görg C . Diagnostic accuracy of B‐mode‐ and contrast‐enhanced ultrasound in differentiating malignant from benign pleural effusions. Diagnostics 2021; 11(7): 1293. 34359376 10.3390/diagnostics11071293 PMC8305637

[19] Metlay JP , Waterer GW , Long AC , Anzueto A , Brozek J , Crothers K , *et al*. Diagnosis and treatment of adults with community‐acquired pneumonia. An official clinical practice guideline of the American Thoracic Society and Infectious Diseases Society of America. Am J Respir Crit Care Med 2019; 200(7): e45–67. 31573350 10.1164/rccm.201908-1581ST PMC6812437

[20] Balk DS , Lee C , Schafer J , Welwarth J , Hardin J , Novack V , *et al*. Lung ultrasound compared to chest X‐ray for diagnosis of pediatric pneumonia: a meta‐analysis. Pediatr Pulmonol 2018; 53(8): 1130–9. 29696826 10.1002/ppul.24020

[21] Ticinesi A , Lauretani F , Nouvenne A , Mori G , Chiussi G , Maggio M , *et al*. Lung ultrasound and chest x‐ray for detecting pneumonia in an acute geriatric ward. Medicine (Baltimore) 2016; 95(27): e4153. 27399134 10.1097/MD.0000000000004153 PMC5058863

[22] Mathis G . Pneumonia: does ultrasound replace chest X‐ray? Praxis (Bern 1994) 2018; 107(23): 1283–7. 30424687 10.1024/1661-8157/a003111

[23] Ewig S , Kolditz M , Pletz M , Altiner A , Albrich W , Drömann D , *et al*. Behandlung von erwachsenen Patienten mit ambulant erworbener Pneumonie – Update 2021. Pneumologie 2021; 75(9): 665–729. 34198346 10.1055/a-1497-0693

[24] Zanobetti M , Scorpiniti M , Gigli C , Nazerian P , Vanni S , Innocenti F , *et al*. Point‐of‐care ultrasonography for evaluation of acute dyspnea in the ED. Chest 2017; 151(6): 1295–301. 28212836 10.1016/j.chest.2017.02.003

[25] Mathis G . Bildatlas der Lungensonographie. Heidelberg, Germany: Springer; 2016.

[26] Mathis G . Chest sonography. Cham, Switzerland: Springer; 2017.

[27] Reissig A , Copetti R , Mathis G , Mempel C , Schuler A , Zechner P , *et al*. Lung ultrasound in the diagnosis and follow‐up of community‐acquired pneumonia. Chest 2012; 142(4): 965–72. 22700780 10.1378/chest.12-0364

[28] Görg C , Bert T , Kring R , Dempfle A . Transcutaneous contrast enhanced sonography of the chest for evaluation of pleural based pulmonary lesions: experience in 137 patients. Ultraschall Med 2006; 27(5): 437–44. 17033945 10.1055/s-2006-927021

[29] Caremani M , Benci A , Lapini L , Tacconi D , Caremani A , Ciccotosto C , *et al*. Contrast enhanced ultrasonography (CEUS) in peripheral lung lesions: a study of 60 cases. J Ultrasound 2008; 11(3): 89–96. 23397023 10.1016/j.jus.2008.05.008 PMC3553306

[30] Safai Zadeh E , Görg C , Prosch H , Jenssen C , Blaivas M , Laursen CB , *et al*. WFUMB technological review: how to perform contrast‐enhanced ultrasound of the lung. Ultrasound Med Biol 2022; 48: 598–616. 35067423 10.1016/j.ultrasmedbio.2021.11.014

[31] Linde HN , Holland A , Greene BH , Görg C . Kontrastunterstützte Sonografie (CEUS) bei Pneumonie: Darstellungsmuster und prognostische Bedeutung ‐ eine retrospektive Studie bei n=50 Patienten [Contrast‐enhancend sonography (CEUS) in pneumonia: typical patterns and clinical value ‐ a retrospective study on n=50 patients]. Ultraschall Med. 2012; 33(2): 146–51. German. 10.1055/s-0031-1273280. Epub 2011 May 31. 21630185

[32] Sidhu P , Cantisani V , Dietrich C , Gilja OH , Saftoiu A , Bartels E , *et al*. The EFSUMB guidelines and recommendations for the clinical practice of contrast‐enhanced ultrasound (CEUS) in non‐hepatic applications: update 2017 (Long version). Eur J Ultrasound 2018; 39(2): e2–e44. 10.1055/a-0586-1107 29510439

[33] Htun TP , Sun Y , Chua HL , Pang J . Clinical features for diagnosis of pneumonia among adults in primary care setting: a systematic and meta‐review. Sci Rep 2019; 9(1): 7600. 31110214 10.1038/s41598-019-44145-y PMC6527561

[34] Görg C , Bert T , Görg K . Contrast‐enhanced sonography for differential diagnosis of pleurisy and focal pleural lesions of unknown cause. Chest 2005; 128(6): 3894–9. 16354860 10.1378/chest.128.6.3894

[35] Kirchner J . Alveolar pneumonia. Chest radiology: A Resident's manual. Stuttgart, Germany: Georg Thieme Verlag KG; 2011. 10.1055/b-0034-74190.

[36] Lo Giudice V , Bruni A , Corcioni E , Corcioni B . Ultrasound in the evaluation of interstitial pneumonia. J Ultrasound 2008; 11(1): 30–8. 23396220 10.1016/j.jus.2007.10.002 PMC3553113

[37] Safai Zadeh E , Beutel B , Dietrich CF , Keber CU , Huber KP , Görg C , *et al*. Perfusion patterns of peripheral pulmonary lesions in COVID‐19 patients using contrast‐enhanced ultrasound (CEUS). J Ultrasound Med 2021; 40: 2403–11. 33459393 10.1002/jum.15624 PMC8014529

[38] Safai Zadeh E , Dietrich CF , Kmoth L , Trenker C , Alhyari A , Ludwig M , *et al*. Peripheral pulmonary lesions in confirmed pulmonary arterial embolism: follow‐up study of B‐mode ultrasound and of perfusion patterns using contrast‐enhanced ultrasound (CEUS). J Ultrasound Med 2022; 41: 1713–21. 34694040 10.1002/jum.15852

[39] Dunham‐Snary KJ , Wu D , Sykes EA , Thakrar A , Parlow LRG , Mewburn JD , *et al*. Hypoxic pulmonary vasoconstriction: from molecular mechanisms to medicine. Chest 2017; 151(1): 181–92. 27645688 10.1016/j.chest.2016.09.001 PMC5310129

[40] Safai Zadeh E , Keber CU , Dietrich CF , Westhoff CC , Günter C , Beutel B , *et al*. Perfusion patterns of peripheral pulmonary granulomatous lesions using contrast‐enhanced ultrasound (CEUS) and their correlation with immunohistochemically detected vascularization patterns. J Ultrasound Med 2022; 41: 565–74. 33955572 10.1002/jum.15730

[41] Safai Zadeh E , Westhoff CC , Keber CU , Trenker C , Dietrich CF , Alhyari A , *et al*. Perfusion patterns of peripheral organizing pneumonia (POP) using contrast‐enhanced ultrasound (CEUS) and their correlation with immunohistochemically detected vascularization patterns. Diagnostics 2021; 11(9): 1601. 34573943 10.3390/diagnostics11091601 PMC8468045

[42] Castro O , Brambilla DJ , Thorington B , Reindorf CA , Scott RB , Gillette P , *et al*. The acute chest syndrome in sickle cell disease: incidence and risk factors. The cooperative study of sickle cell disease. Blood 1994; 84(2): 643–9. 7517723

[43] Findeisen H , Trenker C , Figiel J , Greene BH , Görg K , Görg C . Vaskularisation primärer peripherer Lungenkarzinome in der kontrastmittelunterstützten Sonografie (CEUS) – retrospektive Studie bei n=89 Patienten. Ultraschall Med 2019; 40(5): 603–8. 30332711 10.1055/a-0725-7865

